# Investigation of Personality Traits between Infertile Women Submitted to Assisted Reproductive Technology or Surrogacy

**DOI:** 10.22074/ijfs.2016.4774

**Published:** 2016-04-05

**Authors:** Najmeh Asgari, Fariba Yazdkhasti, Mohammad Hossein Nasr Esfahani

**Affiliations:** 1 Department of Psychology, Faculty of Psychology and Educational Sciences, University of Isfahan, Isfahan, Iran; 2 Isfahan Fertility and Infertility Center, Isfahan, Iran

**Keywords:** Flexibility, Happiness, Assisted Reproductive Technology

## Abstract

**Background:**

Personality traits affect human relationships, social interactions, treatment procedures, and essentially all human activities. The purpose of this study is to investigate the personality traitsincluding sensation seeking, flexibility, and happiness among a variety of infertile women who were apt to choose assisted reproductive technology (ART) or surrogacy.

**Materials and Methods:**

This is a cross-sectional study that was performed on 251 infertile women who visited Isfahan and Tehran Reproductive Medicine Center. These fertility clinics are located in Isfahan and Tehran, Iran. In this study, 201 infertile women who underwent treatment using ART and 50 infertile women who tended to have surrogacy were chosen by convenience sampling. Zuckerman’s Sensation Seeking Scale Form V (SSS-V), Psychological Flexibility Questionnaire (adapted from NEO Personality Inventory-Revised) and Oxford Happiness Questionnaire (OHQ) were used as research instruments. All participants had to complete the research instruments in order to be included in this study. Data were analyzed by descriptive-analytical statistics and statistical tests including multivariate analysis of variance (MANOVA) and Z Fisher. Statistically significant effects were accepted for P<0.05.

**Results:**

In the sensation-seeking variable, there was a meaningful difference between under-study groups. However, the flexibility and happiness variables did not have a significant difference between under-study groups (P<0.001). Interaction between education, employment, and financial status was effective in happiness of infertile women underwent ART (P<0.05), while age, education and financial status were also effective in happiness of infertile women sought surrogacy (P<0.05). A positive meaningful relationship was seen between sensation seeking and flexibility variables in both groups (P<0.05). And a negative meaningful relationship was seen between sensation seeking and happiness in infertile women who sought surrogacy (P<0.05). The difference in rate of relationship between sensation seeking and flexibility was meaningful in infertile women who sought either ART or surrogacy (P<0.05).

**Conclusion:**

Sensations seeking as a personality trait is lower in infertile women who underwent treatment using ART compared women who tended to have surrogacy. This study shows that demographic variables are effective in happiness of infertile women. Also, there is a significant relation among sensation seeking, flexibility and happiness in infertile women.

## Introduction

Infertility is a biological, mental, and social phenomenon; in other words, there are some mental, physiological, environmental, and interactional aspects affecting infertility (1). Infertility is defined as the inability to conceive, despite sexual intercourse, during a year without using any contraceptive devices. It is important to know that this period of time decreases to 6 months for women over 35 as this group of women naturally suffers a decrease in their conception ability (2).

Receiving treatments has increased the conception chance among infertile couples who have lost their ability to conceive (3). However, a research has indicated that a myriad of factors cause tension in these groups, including several medical tests, length of infertility treatment, low level of treatment success, and financial hardship from infertility treatment costs. These factors are so important that they cause infertile individuals to discontinue the treatment (4). Personality traits are among effective factors on human relation, social interaction, treatment procedure, and generally all human activities in the community (5). Sensation-seeking, flexibility, and happiness are three of the most important components of personality traits that can influence an individual’s response to stressful situations.

Sensation seeking is a personality trait that puts emphasis on human behavior and interpersonal relations. As a result, many individual differences can be justified according to this trait. As Zuckerman explained, Sensation seeking is a personality trait which is experienced by seeking feelings and extensive experiences, and by accepting physical, social, legal and financial dangers (6). According to the Zuckerman, sensation seeking is a known trait that occurs with risk-taking (7). Risk-taking behaviors are those behaviors that make physical, psychological, and social results more likely to be negative and destructive (8).

A research has indicated that women who underwent assisted reproductive technology (ART) are at the increased risk of endometrial (9) and ovarian cancer (10-12). Children whose mothers gave birth with the help of ART are also more likely to suffer from physical problems. The study carried out by Ceelen et al. (13) has indicated that the risk of cardiovascular disease is more common in children born with *in vitro* fertilization (IVF). Furthermore in Iran, insurance companies refuse to cover the costs of fertility treatment, so patients have to accept the financial risks.

Surrogacy also has its own dangers; patients undergoing this treatment have to accept the probable risks (14, 15), including: increased risk of infertility, regret after facing surrogacy difficulties (16), refusing to give the baby to the couple, having a relationship with the baby’s father (17), and not caring for the fetal health (18). Thus both ART and surrogacy have a number of risk factors that may be related with sensation seeking of women undergoing these procedures.

Another important personality trait that can affect infertility treatment procedures is flexibility. Psychological flexibility is defined as the ability to "recognize and adapt to various situational demands; shift mindsets or behavioral repertoires when these strategies compromise personal or social functioning; maintain balance among important life domains; and be aware, open, and committed to behaviors that are congruent with deeply held values" (19).

Some studies have demonstrated that flexibility in infertile couples could be an unknown protective factor against regression, resulting from infertility and reduction in life quality (20). In the research carried out by Repokari et al. (21), they have shown that some couples undergoing ART are more flexible to the negative effect of stressful psychological factors and consequently experience a feeling of bonding during infertility treatment.

In the case of surrogacy, flexibility again is a positive aspect of treatment. The American Society for Reproductive Medicine (ASRM) has pointed out that sympathy, compatibility, and flexibility in the couple seeking surrogacy guarantee the treatment success (22).

Happiness appears to be another personality variable. The following is the definition given by Argyle on happiness:

Happiness is the state of being happy or delightful (positive excitements), being satisfied with life being far from any anxiety and depression (negative sentiment) (23). Happiness is within the realm of health psychology and under the effect of different factors such as mental (24), physical (25), economical (26) and religious (27) factors. Therefore, many personal differences can be justified based on these factors.

Research studies have revealed that the quality of marital relationship is considered as a meaningful predictive factor in happiness and desirable life, while low marital quality can lead to a myriad of social and family problems (28). Results of some other research studies, on the other hand, have demonstrated that infertility is associated with marital problems, causing a lot of mental and social problems (29). Therefore, it is hypothesized that infertility leads to low level of happiness in such individuals.

There is a number of research studies on depression and anxiety of infertile women, indicating that these women suffer from a high level of depression and anxiety (30-32). Depression could influence infertility treatment, followup programs, and future hope in these patients (33, 30). According to results from Ferreira's research, happiness increases the probability of continuing follow-up treatment programs in these women (34).

The purpose of this study is to investigate the personality traits including sensation seeking, flexibility, and happiness among a variety of infertile women who were apt to choose ART or surrogacy.

## Materials and Methods

Approval for this cross-sectional study was obtained from the Isfahan University, Isfahan, Iran, in 2013. This study was performed on 251 infertile women who visited Isfahan Reproductive Center and Tehran Royan research Center. These fertility centers are located in Isfahan and Tehran, Iran. In this study, 201 infertile women who underwent ART and 50 infertile women who underwent surrogacy were selected by convenience sampling. Researchers attended these centers 4 days per week from April 13, 2013 to July 7, 2013. All participants were asked to sign an informed consent before entering the research. Data was collected using Zuckerman’s Sensation Seeking Scale Form V (SSS-V), Psychological Flexibility Questionnaire (adapted from NEO Personality Inventory Revised) and Oxford Happiness Questionnaire (OHQ). All participants also had to complete the research instruments in order to be included in this study.

### Zuckerman’s sensation seeking scale form V

This questionnaire was developed by Marvin Zuckerman in 1978. Different studies have estimated the reliability of this questionnaire higher than 0.85. In the study performed by Corulla (35), internal validity of this questionnaire was estimated 0.86 for females and 0.83 for males. In a research carried on university students, Mahvi Shirazi (36) reported the validity and reliability values of sensation seeking questionnaire were 0.78 and 0.80, respectively.

### Psychological flexibility questionnaire

This scale is a collection of questions on three traits of imagination (O1), beliefs (O5) and familiarity factor values (O6), which was adapted from five main factors of personality. Different studies have estimated the reliability of this questionnaire higher than 0.85. In a study by Costa and McCrae (37), the validity of flexibility in the quintuple standard personality questionnaire was reported 0.87. In Iran, reliability coefficient for flexibility questionnaire was estimated 0.80 (38). Other studies in Iran by Keshavarz et al. (39) achieved Cronbach alpha of this questionnaire for 0.70.

### Oxford happiness questionnaire

Argyle et al. (40) developed this scale in 1989. Different studies estimate reliability of this questionnaire about 0.9. A number of studies have been conducted on the validity and reliability of the OHQ by Liaghatdar et al. (41) as well as Alipur and Nurbala (42). They reported a satisfactory internal consistence, indicating that total score of all 29 items yielded high correlation coefficients. The reliability values achieved by Cronbach’s alpha and split-half were 0.93 and 0.92, respectively.

### Statistical analysis

All statistical analyses were performed using the Statistical Package for Social Sciences 19.0 (SPSS, SPCC Inc., USA) software. Data were analyzed by descriptive-analytical statistics and statistical tests including multi-variable analysis of variance (MANOVA) and Z Fisher. Statistically significant effects were accepted for P<0.05.

## Results

In this study, 88.4% of samples were over 40 years of age and 11.6% were below 40. As far as education is concerned, 12.7% of participants did not have their high school diploma, 44.6% had received their high school diploma, 12% had associate degree, 25.5% had a bachelor’s science (B.Sc.), and 5.2% had a master’s degree or higher. Regarding employment status, 74.5% of participants were housewives and 25.5% were employees.

Indexes of descriptive statistics including mean and SD are available in Table 1. In order to analysis of the differences between under-study groups in the field of sensation seeking, flexibility, and happiness, we employed MANOVA, shown in Table 2. The assumption of homogeneity of variances was carried out using Levene’s.

According to Table 2, there are meaningful differences regarding sensation seeking between groups. So the rate of sensation seeking is significantly higher in surrogacy group compared to ART treatment group. However, there are no meaningful differences regarding happiness and flexibility between two groups.

Tables 3 and 4 show the results of MANOVA, indicating the differences created in sensation seeking, happiness, and flexibility variables by participants’ age, education level, employment status, and financial status.

Table 3 depicts the interaction of education, employment, and financial status affects the happiness in infertile women who sought treatment. Moreover, interaction between education and financial status as well as the interaction between employment and financial status affect happiness. However, interaction between education and employment affects the sensation seeking. Table 3 also demonstrates that in ART treatment group, education affects the sensation seeking, employment status affects the flexibility, and financial status affects the happiness.

Table 4 indicates that only age, education, and financial status affects happiness in infertile women who underwent surrogacy.

**Table 1:** Descriptive statistics of groups


Group variable	Sensation seeking			Flexibility			Happiness	
	n	Mean	SD	n	Mean	SD	n	Mean	SD

Seeking treatment	201	20.627	3.749	201	26.91	5.498	201	42.22	12.94
Surrogacy	50	23.24	3.879	50	26.98	5.192	50	39.98	11.54


**Table 2:** Summary of MANOVA


Difference source	Statistical index	SS	df	MS	F	Meaningful level

Intergroup	Sensation seeking	273.411	1	273.411	19.187	0.001*
	Flexibility	0.194	1	0.194	0.007	0.936
	Happiness	201.601	1	201.601	1.254	0.264
Intragroup	Sensation seeking	3548.135	249	14.25		
	Flexibility	7367.368	249	29.588		
	Happiness	40021.905	249	160.731		
Sum	Sensation seeking	116072	251			
	Flexibility	189322	251			
	Happiness	478296	251			


MANOVA; Multi-variable analysis of variance, SS; Sum of squares, df; Degree of freedom, MS; Mean squares, F; Function and *; P<0.001.

**Table 3:** 


Difference source	Statistical index	SS	df	MS	F	Meaningful level

Age	Sensation seeking	16.012	1	16.012	1.4	0.239
	Flexibility	2.437	1	2.437	0.08	0.774
	Happiness	34.27	1	34.27	0.25	0.618
Education level	Sensation seeking	300.15	5	60.031	5.24	0.000*
	Flexibility	58.417	5	11.683	0.4	0.851
	Happiness	1133.1	5	226.61	1.65	0.15
Employment status	Sensation seeking	13.514	1	13.514	1.18	0.279
	Flexibility	224.35	1	224.35	7.62	0.006*
	Happiness	29.385	1	29.385	0.21	0.645
Financial status	Sensation seeking	5.908	2	2.954	0.26	0.773
	Flexibility	9.125	2	4.563	0.16	0.857
	Happiness	4180.9	2	5.831	15.2	0.000*
Interaction between age and education	Sensation seeking	45.379	2	22.69	1.98	0.141
	Flexibility	1.704	2	0.852	0.03	0.971
	Happiness	147.19	2	73.594	0.54	0.587
Interaction between age and employment status	Sensation seeking	0.00	0	_	_	_
	Flexibility	0.00	0	_	_	_
	Happiness	0.00	0	_	_	_
Interaction between age and financial status	Sensation seeking	0.00	0	_	_	_
	Flexibility	0.00	0	_	_	_
	Happiness	0.00	0	_	_	_
Interaction between education and employment status	Sensation seeking	144.27	2	72.135	6.3	0.002*
	Flexibility	55.251	2	27.626	0.94	0.393
	Happiness	42.026	2	21.013	0.15	0.859
Interaction between education and financial status	Sensation seeking	94.956	6	15.826	1.38	0.224
	Flexibility	41.286	6	6.881	0.23	0.965
	Happiness	3096.5	6	516.08	3.75	0.002*
Interaction between employment status and financial	Sensation seeking	0.047	1	0.047	0.00	0.949
	Flexibility	28.763	1	28.763	0.98	0.324
	Happiness	758.39	1	758.39	5.51	0.02*
Interaction between age, education and employment status	Sensation seeking	0.00	0	_	_	_
	Flexibility	0.00	0	_	_	_
	Happiness	0.00	0	_	_	_
Interaction between age, education and financial status	Sensation seeking	0.00	0	_	_	_
	Flexibility	0.00	0	_	_	_
	Happiness	0.00	0	_	_	_
Interaction between age, employment status and financial status	Sensation seeking	0.00	0	_	_	_
	Flexibility	0.00	0	_	_	_
	Happiness	0.00	0	_	_	_
Interaction between education, employment status and financial status	Sensation seeking	11.797	1	11.797	1.03	0.311
	Flexibility	20.076	1	20.076	0.68	0.41
	Happiness	885.92	1	885.92	6.44	0.012*
Interaction between age, education, employment status and financial status	Sensation seeking	0.00	0	_	_	_
	Flexibility	0.00	0	_	_	_
	Happiness	0.00	0	_	_	_
Error	Sensation seeking	2038.4	178	11.452
	Flexibility	5243.4	178	29.458
	Happiness	24505	178	137.67
Sum	Sensation seeking	88330	201
	Flexibility	151605	201
	Happiness	391849	201


MANOVA; Multi-variable analysis of variance, ART; Assisted reproductive technology, SS; Sum of squares, df; Degree of freedom, MS; Mean squares, F; Function and *; P<0.05.

**Table 4:** The summary of the MANOVA of infertile women sought surrogacy


Difference source	Statistical index	SS	df	MS	F	Meaningful level

Age	Sensation seeking	4.316	1	4.316	0.33	0.572
	Flexibility	4.967	1	4.967	0.23	0.636
	Happiness	38.165	1	38.165	0.48	0.494
Education level	Sensation seeking	40.051	5	8.01	0.61	0.695
	Flexibility	88.769	5	17.754	0.82	0.547
	Happiness	167.18	5	33.436	0.42	0.831
Employment status	Sensation seeking	0.018	1	0.018	0.00	0.971
	Flexibility	10.081	1	10.081	0.46	0.501
	Happiness	116.67	1	116.67	1.47	0.236
Financial status	Sensation seeking	34.458	2	17.229	0.26	0.286
	Flexibility	8.812	2	4.406	0.2	0.817
	Happiness	96.165	2	48.083	0.61	0.553
Interaction between age and education	Sensation seeking	11.836	3	3.945	0.3	0.826
	Flexibility	1.704	3	1.584	0.07	0.974
	Happiness	147.19	3	52.255	0.66	0.585
Interaction between age and employment status	Sensation seeking	0.00	0	_	_	_
	Flexibility	0.00	0	_	_	_
	Happiness	0.00	0	_	_	_
Interaction between age and financial status	Sensation seeking	0.06	1	0.06	0.01	0.947
	Flexibility	1.294	1	1.294	0.06	0.809
	Happiness	108.35	1	108.35	1.36	0.253
Interaction between education and employment status	Sensation seeking	0.159	1	0.159	0.3	0.913
	Flexibility	37.398	1	37.398	1.72	0.2
	Happiness	128.14	1	128.14	1.62	0.214
Interaction between education and financial status	Sensation seeking	101.09	2	50.543	3.83	0.033
	Flexibility	46.476	2	23.238	1.07	0.356
	Happiness	224.14	2	112.07	1.41	0.261
Interaction between employment status and financial	Sensation seeking	0.00	0	_	_	_
	Flexibility	0.00	0	_	_	_
	Happiness	0.00	0	_	_	_
Interaction between age, education and employment status	Sensation seeking	0.00	0	_	_	_
	Flexibility	0.00	0	_	_	_
	Happiness	0.00	0	_	_	_
Interaction between age, education and financial status	Sensation seeking	26.501	1	26.501	2.01	0.167
	Flexibility	1.726	1	1.726	0.08	0.78
	Happiness	449.23	1	449.23	5.65	0.024*
Interaction between age, employment status and financial status	Sensation seeking	0.00	0	_	_	_
	Flexibility	0.00	0	_	_	_
	Happiness	0.00	0	_	_	_
Interaction between education, employment status and financial status	Sensation seeking	0.00	0	_	_	_
	Flexibility	0.00	0	_	_	_
	Happiness	0.00	0	_	_	_
Interaction between age, education, employment status and financial status	Sensation seeking	0.00	0	_	_	_
	Flexibility	0.00	0	_	_	_
	Happiness	0.00	0	_	_	_
Error	Sensation seeking	382.5	29	13.19
	Flexibility	629.69	29	21.714
	Happiness	2306.3	29	79.528
Sum	Sensation seeking	737.12	50
	Flexibility	1321	50
	Happiness	6527	50


MANOVA; Multi-variable analysis of variance, SS; Sum of squares, df; Degree of freedom, MS; Mean squares, F; Function and * ; P<0.05.

Fisher’s z was used to study the meaningful difference between rates of the correlations in ART treatment and surrogacy groups. In this statistical method, r coefficients are changed to z scores and then differences between z scores are computed. Achieved results are available in Tables 5 and 6. In Table 5, the transformation of Fisher’s r into z-scores is shown.

Table 6 shows the meaningful differences between correlations of two groups and also it shows only the difference in correlation between sensation seeking and flexibility is significant. Based on Table 5, this correlation is significantly higher in surrogacy group compared to ART treatment group.

**Table 5:** Transformation of Fisher’s r into Z-scores


Group	The correlation between sensation seeking and flexibility	The correlation between sensation seeking and happiness	The correlation between flexibility and happiness	Changing r into Z-scores for sensation seeking and flexibility	Changing r into Z-scores for sensation seeking and happiness	Changing r into Z-scores for flexibility and happiness

Group 1	0.206*	0.00	0.039	0.011	0.00	0.333
Group 2	0.394*	-0.321*	-0.258	0.375	0.039	0.264


*; P<0.05.

**Table 6:** The meaningful differences between correlations of two groups


Sensation seeking Z and flexibility in both groups	Sensation seeking Z and happiness in both groups	Flexibility Z and happiness in both groups

-2.25*	-0.24	0.94


*; P<0.05.

## Discussion

The results of this research study provide families, therapists and mental health counselors, researchers, and infertile women with many practical applications. Understanding personality type of infertile woman can help her to choose a proper treatment method. Infertility is a phenomenon that therapists need to be aware of and skillful enough to prevent the tensions treating physical and mental health of infertile women.

According to the achieved results, there was a meaningful difference in terms of sensation seeking between groups. However, the same story was not true for flexibility and happiness. The researchers couldn't find a similar research that had considered these variables with the same groups. Although there are a number of studies pointing out the following risks that a person underwent surrogacy may experience (43): social risks; psychological risks including remorse (16), not withholding the baby, having a relationship with the baby’s father (17) and not caring for the fetal health (18, 44); legal risks (45); financial risks (46, 47, 14, 15) as well as the potential risks of ART (43). However, our finding indicated high level of sensation seeking in a large number of individuals who underwent surrogacy.

According to Zuckerman (1979 and 1991), education, employment, and financial status affect the sensation seeking of infertile women underwent treatment. He showed that participants with university degrees achieved a higher score in sensation seeking. Moreover sensation seeking, adventure seeking, and experience seeking were seen among individuals appertaining to middle or high class of society (48, 49).

According to other findings, employment status can affect flexibility in infertile women who sought treatment. As Bradbury et al. (50) suggested a variety of factors can affect the marital compatibility and individuals’ flexibility. Similar to their findings, we pointed out the sociocultural variables such as age, education, employment status, and financial status as effective factors.

Concerning the effect of demographic features on infertile women’s happiness, our results indicated that financial status and education affected the happiness in infertile women who underwent treatment. Interaction between age, education and financial status also affected the happiness in infertile women who sought surrogacy. These findings were also in agreement with Ramezanzadeh et al. (51) and De Ree and Alessie (52). According to the results achieved by Ramezanzadeh et al. (51), education level causes a decrease in infertile women’s depression, while it leads to an increase in happiness in them. Thus, education plays an important role in generating interests and developing self-satisfaction, resulting in happiness. Additionally, the relationship between education level and happiness is mostly resulted from high education level, successful career and high income (52).

According to achieved results, there was a positive meaningful relation between sensation seeking and flexibility in both groups of infertile women, indicating the meaningful difference regarding rates of relationships in the two groups. These findings were also in agreement with Lauriola and Levin (53), Nicholson et al. (54), Soane and Chmiel (55), Anic (56) and Vries et al. (57). Sensation-seeking individuals are the most willing to take risks. They often seek new experiences and are willing to challenge possibilities. Sensationseekers do not hesitate to go in different directions, especially when it comes to new ideas and innovations. They also have a high tendency to adapt quickly to changing circumstances because it feeds their desire for novel experiences. Therefore, flexibility is expected to have positive relationship with sensation seeking (54).

There were no meaningful differences between groups concerning the relationship between flexibility and happiness. Thus, flexible individuals increase their happiness because they own more choices (58). These findings are in agreement with those of Hayes and Joseph (59). However, since women who sought treatment failed many times in their programs, they may experience negative sensational feelings, suggesting no meaningful relation between flexibility and happiness.

Concerning the relationship between sensation seeking and happiness, a significant negative relationship is observed among infertile women seeking surrogacy. To shed light on these results, it should be noted that women seeking surrogacy, due to their specific conditions, must undergo a hazardous and costly treatment. The difficulties of these treatments lead to a reduction of overall happiness. However, this relationship is not observed in women undergoing ART. It is recommended this issue to be considered in future studies where personality traits can have a meaningful relation with infertile women’s preferences over the treatment method. Another recommendation is to develop interference programs for recognizing personality traits and increasing flexibility and happiness in the group of infertile women, so women can respond to the pressures resulting from infertility and treatment more easily and make better decisions on treatment methods. One of the limitations of this study was the low number of women seeking surrogacy. Furthermore we only investigated three traits of personality traits, while other personality traits may be important to the subject of this study. Also a cross sectional study does not allow causeeffect conclusions, so the results of the study had no control over the confounding variables.

## Conclusion

Based on the results of this study, sensations seeking as a personality trait is lower in infertile women who underwent ART as compared women who tended to have surrogacy. This study showed that demographic variables were effective in happiness of infertile women. Also there is a significant relation among sensation seeking, flexibility and happiness in infertile women. Thus in infertile women, those with higher rates of sensation seeking were more likely to choose surrogacy rather than ART treatment, and these individuals needed further psychological intervention to improve their happiness.
